# “My children and I will no longer suffer from malaria”: a qualitative study of the acceptance and rejection of indoor residual spraying to prevent malaria in Tanzania

**DOI:** 10.1186/1475-2875-11-220

**Published:** 2012-07-02

**Authors:** Michelle R Kaufman, Datius Rweyemamu, Hannah Koenker, Jacob Macha

**Affiliations:** 1 Johns Hopkins Bloomberg School of Public, HealthCenter for Communication Programs, 111 Market Place Suite 310, Baltimore, MD, 21202, USA; 2 Department of Sociology, University of Dar es Salaam, Dar es Salaam, P.O. Box 35043, Tanzania; 3 Johns Hopkins Bloomberg School of Public Health, Center for Communication Programs, Kimweri Road Msasani, Dar es Salaam, PO Box 105303, Tanzania

**Keywords:** Indoor residual spraying, Tanzania, Insecticide, Maneno muhimu, Upuliziaji wa dawa ya kuua mbu majumbani, Tanzania, Dawa

## Abstract

**Background:**

The objective of this study was to identify attitudes and misconceptions related to acceptance or refusal of indoor residual spraying (IRS) in Tanzania for both the general population and among certain groups (*e.g.*, farmers, fishermen, community leaders, and women).

**Methods:**

This study was a series of qualitative, semi-structured, in-depth interviews and focus group discussions conducted from October 2010 to March 2011 on Mainland Tanzania and Zanzibar. Three groups of participants were targeted: acceptors of IRS (those who have already had their homes sprayed), refusers (those whose communities have been sprayed, but refused to have their individual home sprayed), and those whose houses were about to be sprayed as part of IRS scale-up. Interviews were also conducted with farmers, fishermen, women, community leaders and members of non-government organizations responsible for community mobilization around IRS.

**Results:**

Results showed refusers are a very small percentage of the population. They tend to be more knowledgeable people such as teachers, drivers, extension workers, and other civil servants who do not simply follow the orders of the local government or the sprayers, but are skeptical about the process until they see true results. Refusal took three forms: 1) refusing partially until thorough explanation is provided; 2) accepting spray to be done in a few rooms only; and 3) refusing outright. In most of the refusal interviews, refusers justified why their houses were not sprayed, often without admitting that they had refused. Reasons for refusal included initial ignorance about the reasons for IRS, uncertainty about its effectiveness, increased prevalence of other insects, potential physical side effects, odour, rumours about the chemical affecting fertility, embarrassment about moving poor quality possessions out of the house, and belief that the spray was politically motivated.

**Conclusions:**

To increase IRS acceptance, participants recommended more emphasis on providing thorough public education, ensuring the sprayers themselves are more knowledgeable about IRS, and asking that community leaders encourage participation by their constituents rather than threatening punishment for noncompliance. While there are several rumours and misconceptions concerning IRS in Tanzania, acceptance is very high and continues to increase as positive results become apparent.

**Swahili Abstract:**

**Usuli:**

Malengo mahususi ya utafiti huu ni kutambua tabia na imani potofu zinazopelekea kukubali au kutakaa upuliaziaji wa dawa ya kuua mbu majumbani (IRS) katika Tanzania kwa watu wote kwa ujumla na kwa makundi maalumu ya watu (kama wakulima, wavuvi, viongozi wa jamii na wanawake).

**Njia:**

Utafiti huu ni mfululizo wa tafiti stahilifu zenye sehemu ya muundo, tafiti za kina na majadilianao ya vikundi vya walengwa yaliyofanyika Tanzania bara na Zanzibar kuanzia mwezi Oktoba, 2010 hadi mwezi Machi, 2011. Yalikuwepo makundi matatu ya walengwa: wanaokubali IRS (wale ambao nyumba zao zilikwisha kupulizwa dawa ya kuua mbu) wasiokubali (hii ni jamii iliyokwisha kupulizwa dawa na wale watu waliokataa dawa isipulizwe kwenye nyumba zao) na wale ambao nyumba zao zilikuwa zinakaribia kupulizwa dawa ikiwa ni kama sehemu ya kusambaza IRS. Usaili ulifanyika pia kwa wakulima, wavuvi, wanawake na viongozi wa jamii vile vile na kwa wanachama wa asasi zisizo za kiserikali waliokuwa wakiwajibika kwa IRS.

**Matokeo:**

Matokeo yalionyesha kuwa waliokataa walikuwa ni asilimia ndogo sana ya watu wote. Walikuwa ni watu waelewa kama vile walimu, madereva, wafanyakazi katika miradi na watumishi wengine wa serikali ambao wanafuata amri kutoka kwa serikali yao au kwa wapuliza dawa lakini walikuwa na wasiwasi kuhusu mchakato huo mpaka waone matokeo yake. Waliokataa walikuwa katika maainisho matatu: 1) waliokataa kidogo mpaka wapewe maelezo; 2) waliokubali dawa ipulizwe kwenye vyumba vichache tu; 3) waliokataa katu katu. Mara kwa mara wengi wa wasailiwa waliokataa, walitoa sababu zao za kukataa nyumba zao zisipuliziwe, bila kukubali kuwa wamekataa kupuliziwa. Sababu za kukataa mwanzoni zilikuwa ni pamoja na; kutokuwa na uhakika kuhusu dawa inavyofanya kazi, kutoelewa matokeo yake, kuongezeka kwa kuenea kwa wadudu wengine. Athari nyingine mbaya zilizoonekana ni: harufu, tetesi kuhusu kemikali zinazoathiri urutubishwaji, aibu ya kutolewa vitu vyao vyenye thamani duni kutoka kwenye nyumba zao na imani kuwa dawa hiyo ilihamasishwa kisiasa zaidi.

**Hitimisho:**

Ili kuongeza kukubalika kwa IRS, washiriki wanasisitiza zaidi kuzitoa dawa hizo kwa kuwaelimisha watu kwanza, kuhakikisha kuwa wanaonyunyuza dawa hiyo wana ujuzi wa kutosha kuhusu dawa yenyewe, kuwaomba viongozi wa jamii wawatie moyo wanajamii katika kaya zao badala ya kuwatishia na kuwalazimisha. Pamoja na kwamba kuna tetesi na watu kuelewa visivyo kuhusu IRS, kukubalika ni kukubwa na kunaendelea kuonyehsa kuwa na mafanikio chanya.

## Background

Indoor Residual Spraying (IRS) is the spraying of the interior of homes with insecticides to kill mosquitoes in order to control malaria on a large scale. IRS has been used to help eliminate malaria from large areas of Asia, Europe, Latin America, and part of Africa. IRS was used in Tanzania in the late 1950s under the Pare-Taveta project in Northeast Tanzania, and in Zanzibar from 1958 to 1968 and from 1981–1987 [1]. The President’s Malaria Initiative (PMI) funded the latest rounds of spraying in Zanzibar starting in 2006 and on the mainland in 2007 in Kagera region, adding Mara and Mwanza regions in 2010. ICON (lambda-cyhalothryn, a pyrethroid) is used under the latest initiative. DDT is no longer registered for use in Tanzania, and there has been some documentation of DDT-resistance [1,2].

The non-profit organization RTI International is currently responsible for scaling up IRS in three regions of mainland Tanzania (Kagera, Mwanza, and Mara), with continued spraying in Zanzibar, under funding from USAID. This programme involves blanket spraying (covering 90 + % of eligible structures), targeted spraying (covering 50% of eligible structures), and focal spraying (responding to “hot-spot” outbreaks). The main objectives of this programme are: 1) scale-up IRS on mainland and maintain high IRS coverage in Zanzibar; 2) conduct epidemic detection and focal-spraying response; 3) develop an environmental compliance strategy and monitoring plan for the mainland and Zanzibar; and 4) establish a viable and sustained entomological monitoring system on the mainland and Zanzibar.

### Malaria in Tanzania

With nearly all of the 41 million residents on the Mainland and all 1.2 million in Zanzibar at risk of malaria [3], Tanzania has the largest number of persons at risk among all 17 countries in the President’s Malaria Initiative [3]. Estimated annual malaria deaths as of 2008 were 87 per 100,000 for the overall population [4]. There are 14–18 million episodes of malaria annually in Tanzania, constituting the largest burden of any disease on government resources [3]. Over 40% of all outpatient attendances are attributable to malaria [3]. According to the Ministry of Heath and Social Welfare (MOHSW) health management information system, the disease is responsible for more than half of all deaths among children under five years of age in health facilities, and up to one-fifth of deaths among pregnant women [3].

Financial costs of malaria in Tanzania cannot be accurately estimated, but adults lose one to five days of work per incident depending on the severity and whether or not they are hospitalized. Relatedly, caretakers lose at least one day of work to care for sick children. In addition, there are cost burdens for treatment and transport to clinics. Malaria also has adverse effects on school attendance and learning ability for children [5].

In Zanzibar, however, the most recent Tanzania HIV/AIDS and Malaria Indicator Survey [6] showed that malaria prevalence stood at 0.8%. In 2010, less than 2% of blood smears from patients at the 90 health facility malaria surveillance sites in Zanzibar were positive for malaria parasites [3]. The decline in incidence is attributed to key interventions, including the use of bed nets, the availability of drugs, environmental cleaning, and the utility of IRS.

### Current utilization of IRS in Tanzania

PMI is currently supporting IRS activities on the Mainland in order to curb malaria incidence and on Zanzibar in order to sustain the low prevalence. On Mainland, IRS was launched in 2007 in Muleba and Karagwe districts, located in Kagera Region. The two districts are located in the Lake Zone, on the shores of Lake Victoria, and are characterized as stable transmission with seasonal variation. Prior to the fieldwork of this study, Muleba and Karagwe successfully implemented five and four rounds of IRS, respectively. IRS activities expanded to the remaining five districts of Kagera Region in 2009, and in 2010 and 2011 to all 18 districts of Kagera, Mwanza, and Mara, covering 1.1 million structures and protecting nearly 6.3 million people [7]. Acceptance rates were 95% [8]. The three rounds of IRS on Mainland have significantly reduced malaria prevalence, hospital admissions, and deaths attributable to malaria.

Since PMI started in 2006, Zanzibar has received six rounds of universal IRS to date, plus blanket spraying covering 85% of eligible structures, targeted spraying (75% of eligible structures), and focal spraying responding to “hot-spot” outbreaks. In the fifth round of spraying, 186,046 (88% of target) were sprayed and protected over a million people. IRS in Zanzibar has significantly contributed to reducing malaria prevalence to less than 1%, enabling Zanzibar to advance to a pre-elimination phase. The sixth round of spraying for Zanzibar occurred in January-February 2010 and targeted eight rural districts [3].

The PMI target is to cover greater than 85% of the households with IRS in order to achieve community coverage sufficient to interrupt transmission of malaria. RTI has consistently met or exceeded this 85% target, but there remains a small percentage of the population in any given district who refuse to allow spray teams into their homes. As the programme expands into new districts, steps must be taken to ensure that households are adequately and appropriately informed of the benefits of IRS to ensure community coverage. As the programme moves from blanket spraying to target spraying and focal spraying, steps must be taken to tailor communication on the process to ensure the targeted coverage levels.

### Addressing IRS concerns in Tanzania

When enacting a large-scale new measure such as IRS to curb incidence of disease, Gramiccia [9] suggests drawing up a health education plan that recognizes community priorities and has inputs from an epidemiologist, sociologist, and health educator working together. The health education staff needs to be continuously supported through training and feedback throughout the life of the project.

The current qualitative study sought to assess the barriers and facilitators to accepting IRS in various communities within Tanzania in the context of current community mobilization techniques. The goal was to use this research to further tailor behavioral change communication (BCC) to continuously increase uptake of the spraying. By addressing the concerns felt by community members who have rejected spraying in the past and to capitalize on the reasons most have accepted the exercise, it is anticipated that the programme can achieve an optimal level of success.

This study focused on knowledge level regarding IRS, attitudes towards the prevention method and malaria, as well as how communities view the ways in which IRS is being rolled out. In addition, it sought to understand, particularly in mainland regions, the misconceptions about IRS that occur within certain groups, including farmers, fishersmen and women.

## Methods

This research was a series of qualitative, semi-structured, in-depth interviews (IDIs) and focus group discussions (FGDs) conducted in three regions of the Lake Zone on mainland Tanzania and Zanzibar. Data was collected from October 2010 through March 2011 by a team of four research facilitators trained on the study protocol and ethical treatment of human participants. Ethical approval for the study was obtained by both the Johns Hopkins University Institutional Review Board in Baltimore, Maryland in the U.S. and the National Institute for Medical Research (NIMR) in Dar es Salaam, Tanzania.

Three groups of participants were targeted for interviews: acceptors of IRS (those who have already had the exercise completed in their homes), refusers (those whose communities have been sprayed, but they refused their individual home to be included), and those whose houses were about to be sprayed as part of the IRS scale-up. FGDs were also conducted with women, farmers, fishers, and community leaders to see if these groups could offer unique insights not captured in the IDIs. Community leaders included village chairpersons (Lake Zone) and *Sheha* committee members (Zanzibar). Table 1 shows the category of participants for each region (acceptors, refusers, about to be sprayed).

**Table 1:** IDI and FGD participants by district and participant selection criteria

**Study Site**	**Number of IDIs (n = 76)**			**Number of FGDs (total n = 57)**			
	***Acceptor***	***Refusers***	***To be sprayed***	***Fishermen***	***Women***	***Farmers***	***Community Leaders***
*Kagera*	8	9	0	0	1	1	0
*Mara*	5	16	2	1	1	0	1
*Mwanza*	7	10	4	0	1	0	0
*Zanzibar*	5	10	0	0	1	0	1
**Total**	**25**	**45**	**6**	**1**	**4**	**1**	**2**

### Participants

All participants were over 18 years of age, with a mean of 45.17 years (range 19–85 years). A majority had only completed primary school. Only two participants received education above the secondary level. Table 2 shows the demographic breakdown of all participants. Table 3 shows the demographic information for refusers only.

**Table 2:** Gender and education level across all participants

Education level*	**Gender**		**Total**
	***Males n(%)***	***Females n(%)***	
*None*	4 (5.7)	6 (8.7)	10 (7.2)
*Primary*	45 (64.3)	51 (73.9)	96 (69.1)
*Secondary*	20 (28.6)	11 (15.9)	31 (22.3)
*Above secondary*	1 (1.4)	1 (1.4)	2 (1.4)
**Total**	**70(100)**	**69(100)**	**139(100)**

Education level was not reported for two participants.

**Table 3:** Characteristics of refusers

	**Gender**		**Total**
	***Male n(%)***	***Female n(%)***	
*Age*			
Below 20 years	0	0	0
20-29 years	4 (16.7)	3 (14.3)	7 (15.5)
30-39 years	4 (16.7)	10 (47.6)	14 (31.1)
40-49 years	5 (20.8)	4 (19.0)	9 (20.0)
50+ years	11 (45.8)	4 (19.0)	15 (33.3)
*Education level*			
None	2 (8.3)	2 (9.5)	4 (8.8)
Primary	18 (75.0)	17 (81.0)	35 (77.7)
Secondary	3 (12.5)	2 (9.7)	5 (11.1)
Above secondary	1 (4.2)	0	1 (2.2)

### Procedures

Participants were selected from Mwanza, Mara, Kagera, and Zanzibar regions. In Mwanza and Mara, Magu and Musoma Rural districts, respectively, were targeted because IRS was in the process of being conducted for the first time in these communities. Kagera region was selected because it was the only region in Mainland Tanzania where IRS had already been ongoing, and during the time of data collection was in its fifth round of spraying. Bukoba Rural District in Kagera Region was purposively selected because the district had a relatively high rate of IRS refusal compared to other districts in the region. In each of the districts, one to two wards were selected for recruitment of participants.

Unlike the Mainland, IRS in Zanzibar has been ongoing for several years and has shown a high rate of success [3]. During the time of data collection, Zanzibar was in its sixth round of spraying. Urban District in Unguja was purposively selected for this study based on epidemiological data on malaria prevalence, patterns of refusal/acceptance of IRS within the past five rounds (2006–2010), and number of insecticide treated nets (ITNs) per sleeping space in the household. According to the preliminary 5^th^ Round Zanzibar IRS Evaluation Report, the Urban District has the lowest proportion of households sprayed (78%), leaving about 6,444 households at risk of malaria transmission.

Individual interviews (n = 76) and 8 FGDs (total n = 57) were conducted in the four regions. IDIs were conducted with the head of the households in participants’ homes in Swahili. FGDs were conducted in a community centre with six to 10 participants per group and were led by a Swahili-speaking facilitator.

Participants were read a consent form in Swahili and gave oral agreement to participate in the study. Participants in the FGDs completed the consent process individually in a private location. IDI and FGD questions focused first on general understanding of malaria and interventions, particularly IRS. The second section focused on reasons why someone might accept or reject IRS for preventing malaria. The interviews wrapped up with discussions of specific IRS rumours in the given community.

IDIs and FGDs were audio recorded, transcribed verbatim, and translated into English. A coding list for themes of interest was developed. Analysis was conducted by two independent coders using Atlas.ti software until at least 80% agreement was reached on the established codes.

## Results

### General knowledge about and attitudes towards malaria

Knowledge of and attitudes towards malaria lay the groundwork for acceptance or rejection of malaria interventions, IRS included. A majority of participants showed basic knowledge of malaria, including correct association between malaria and mosquito bites, its potential fatal consequences, and correct treatment practices. A majority of participants were also able to list several symptoms of malaria, including severe headache, fever, stomachache, dizziness, and vomiting. Correct knowledge of the mechanics of malaria transmission varied by region, however. There were fewer cases of misconceptions about causes and treatment of malaria in Zanzibar compared to other study sites. In some areas, such as Musoma Rural and Magu, several cases of self-treatment and wrong treatment were reported. For instance, when asked what they usually do when they experience symptoms of malaria, some respondents confused proper malaria preventive and treatment measures with other diseases.

### IRS knowledge levels and attitudes towards its effectiveness

Knowledge about IRS is mixed. A substantial number of participants correctly associated malaria, mosquito bites, and IRS. Most participants were aware of what needed to be done before the spray team’s arrival, as well as what the spray teams would do during their visit. However, participants did not know how the insecticide works, the after effects, or what would happen further down the road with continuous spraying. If residents were not present for village meetings during which the process was explained and/or did not receive an informational flyer, they tended to know very little about the exercise. A typical description of the preparation process by those who were knowledgeable was as follows:

"One has to clean all his or her utensils, and all the things need to be taken outside. The house has to be cleaned…That is when they start doing the spraying exercise. When the exercise is done they tell us when we are allowed to return our things inside. (IDI/male/refuser/Bukoba Rural)"

Most of the refusers interviewed were very knowledgeable about how spraying is done and how IRS works. So it is not a lack of knowledge of procedures that led to lack of participation.

A majority of those interviewed reported being happy with the results of IRS and the fact that something is being done to control malaria.

"*Many of my relatives are so thankful about the spraying of the insecticides because since the years this health service started, many people realize that the insecticide is helping us. Our children can’t get malaria fever, which was a big problem to our families due to death of children below one year age up to five years. Therefore, we are very thankful. And we are also saying that if this health will continue we can see how our children are continuing growing in a good health and proceeding well with their studies, as well as pregnant women at home. (FGD/women/Musoma Rural)*"

"*We are thanking our almighty God because now the rate of malaria infections is decreasing. (IDI/female/refuser/Urban District, Zanzibar)*"

In addition, the demand for IRS was higher among those whose houses were sprayed in the previous rounds because participants immediately saw the benefits and were not as skeptical about the outcomes of the exercise. A man interviewed who had accepted during the first round (and was not present during the second round and so was labeled a refused) explained how his house was now free of mosquitoes:

"*To my view I strongly believe that this ICON that is used serves the purpose of its use. For right after the spraying was done, mosquitoes were minimized to a greater extent. For example, from 7 pm up to 9 pm I usually leave my house door open, and the mosquitoes are not seen as before. (IDI/male/refuser/Bukoba Rural)*"

However, some participants were still ambivalent towards the exercise—they saw the benefits of children not getting sick and a lower prevalence of mosquitoes, but there was still a level of skepticism, especially among those who continued to see other insects after the spraying took place.

"*Some [people] are thankful, but people have different views. Others say it’s not good. It [IRS] doesn’t kill even flies. Let’s say like rats, cockroaches are flying. Others are saying it kills all flies. Everyone has his/her own views. But for me all flies have perished.****(****IDI/female/acceptor/Magu)*"

"*Some people are complaining that the spray wasn’t mixed as it was supposed to because when you enter in some houses you feel like itching, and in some houses you won’t feel anything. So people don’t trust that it can kill the mosquitoes. (IDI/male/refuser/Musoma Rural)*"

"*At the moment I heard the sprayed chemicals’ aim is to kill all types of insects and even bats….mice, cockroaches, and all insects in the house. And other people are saying that only cockroaches died….because you can’t see the mosquito….so it’s cockroaches. But these bats didn’t die, mice don’t die. But they said it kills so I don’t understand…. (IDI/male/acceptor/Magu)*"

Another area of confusion involved why spraying was taking place during the dry season when the mosquitoes are not as prevalent as in the rainy season. Also, several people stated they want to know more about the long-term side effects of the spray, especially in those areas where spraying had only occurred once. Since the data for this study were collected during the dry season, some respondents who only received the first round of spray recently or would receive it in the near future understandably stated that they would be better able to comment on what they thought of it during the rainy season when mosquitoes are more prevalent.

Some of those who supported the spraying were so ecstatic that they believed those who refused should be forced to have it done to protect others in the community from malaria:

"*We think something has to be done to them [those who refuse] as a punishment. This is [a] government chemical, and the government can’t bring something to kill the citizens. It is brought to reduce diseases. If he refused, the leaders [of the village] have to take action. (IDI/male/acceptor/Bukoba Rural)*"

A few participants were worried that if their homes were sprayed but their neighbours’ were not, that the spray would not be effective and their families would still suffer.

### Reasons for refusal during the most recent round of spraying

**Figure 1:**
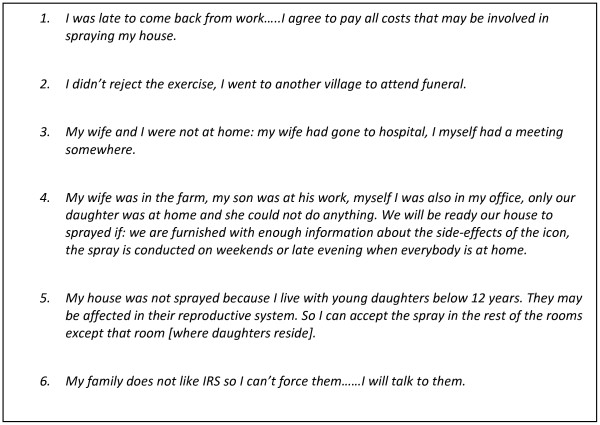
Letters from Kagera residents to community leaders describing reasons for refusal of IRS.

Refusers make up a very small percentage of the targeted households: 5% according to the 2009–10 DHS [8]. They tend to be more knowledgeable people such as teachers, drivers, extension workers, and other civil servants who do not simply follow the orders of the local government or the sprayers but are skeptical about the process until they see true results. Refusal took three different forms: 1) refusing partially until thorough explanation is provided; 2) accepting spray to be done in a few rooms only; and 3) refusing outright. In most of the interviews, refusers justified why their houses were not sprayed, often without admitting that they had refused. Figure 1 shows excerpts from letters written by refusers in Kagera to their community leaders describing why their homes were not sprayed, which was given to the fieldworkers when interviewing the community leader. The reasons why IRS might be refused is outlined in detail below.

#### Initial ignorance

Some community members initially refuse, until they see their neighbours receiving benefits from the spray without side effects, and then opt to accept. Other people do not know enough about the spraying process due to absence during information, education and communication (IEC) or not receiving the flyer explaining the process. However, once they receive more information, most people generally accept it.

"*They [community mobilizers] didn’t tell us anything. For example our kitongoji chairman, if you spy at him, he even doesn’t know what are the reasons [for accepting]. (IDI/Male/to be sprayed/Magu)*"

"*Those who refused, I think they just lack enough information. If they be informed well about the process there will be no problem. There are some people who are hard to understand and to note, but those with easy understanding, they did note it and agreed to the exercise. So for the hard headed it is important that they get education first. I would like the leaders…[to] better follow them [those who refused] to explain to them more regarding this issue of spraying. Because now you may find that a person refuses without any basic reason. But if one…that person is educated well, I think they may come to agree. I would like you [those conducting IRS] to emphasize on providing enough education to us. Let’s not escape from our houses due to being scared of insecticide spraying. (FGD/Women/Musoma Rural)*"

"*In short we didn’t get any details from the leaders…no education. And this is the main obstacle to the exercise—people were forced without education. (IDI/Male/refuser/Musoma Rural)*"

Had community mobilization and IEC been more comprehensive and far-reaching, it is possible that refusals would be even fewer than they are currently.

#### Uncertainty about effectiveness

Another key reason for refusal was uncertainty as to whether or not the process would, indeed, be effective in killing mosquitoes, as explained above. This ineffectiveness could be due to the capacity of the chemical itself or to it being diluted too much by the sprayers:

"*The big problem to those who are already sprayed is seeing the mosquitoes flying and disturbing in one way or another. They think the mixing was not done correctly. (IDI/male/refuser/Musoma Rural)*"

In Zanzibar, where spraying has been done several times and malaria infections have dropped below 1%, the respondents’ concern (both acceptors and refusers) was the ability to maintain the achieved success*.* Without continued education about the benefits of repeated rounds of IRS, it is possible people will no longer find it necessary to have their homes sprayed.

#### Prevalence of other insects

Consistent with research in other countries, there were also concerns about other insects (bed bugs, flies, or cockroaches) that did not disappear or appeared suddenly as a result of the spray:

"*The problem [of IRS] was that after a month since the spraying process was done lice erupted. Thus, everyone started to shy away from this whole process. Even if they were to come to me, I will refuse just like how other people did. Some people said they would close the doors of their houses. (IDI/Female/Refuser/Bukoba Rural)*"

"*What I heard is that this chemical has brought lices and worms. Now I didn’t understand there, meaning that when you look at a fumigated house you see lices and worms appearing in the kitchen. Now that I didn’t understand. (IDI/Male/Refuser/Bukoba Rural)*"

However, this rumour about additional insects appearing varied by study site, depending on the number of IRS rounds. Where there were many rounds of spraying already complete, people were able to provide explanations for such outbreaks of insects, such as animals bringing them into the home, or they saw insects such as lice in houses that were both sprayed and not sprayed.

#### Reported side effects

Complaints about IRS were minimal, but tended to focus on reported side effects. Complaints about itching and/or rash were the most common, followed by stomach upset. But these reports were among a minority of participants. There were also beliefs that the chemical would harm children if they touched the walls, possibly killing them after a long period of exposure. Some believed that if anyone in the household ate something touched by the insecticide, they would become ill. Other side effects included damage to internal organs, swelling of the face and/or body, and the death of any animals (such as hens) that may be living in or near the home.

"*Because back then we had heard they [the spray] have effects. And those effects are caused by what? by those chemicals…So it scares us. Then when we heard that if you fumigate that it is at home, especially in rooms, you may end up with effects. So we were scared of that. (IDI/Female/refuser/Magu)*"

Seeing those conducting the spray process dressed in protective gear while they worked also made some respondents nervous about side effects:

"*What I would like to know is if the chemical has any effects or how doesn’t have the effects as we see the sprayers covered their bodies? We feared....why they don’t leave any part of the body open? The nose is covered, head, legs and even in hands they wear gloves. So we get feared because even the water is soft—it cannot damage anything. Why they don’t cover by a piece of cloth? (laughter) He put on the helmet so the chemical must have the effects or…? So we need to be told the details prior to the exercise. Why did they have to wear that helmet? (IDI/Male/Refuser/Magu)*"

#### Odour

The odour created by the spray was also mentioned by a couple of participants, but this did not seem to be a major concern:

"*Eeeeeeheee, the chemical had a smell! Eee, it really had a smell! But that smell was gone [faded], and it didn’t really harm any human being. (IDI/Female/Acceptor/Bukoba Rural)*"

#### Rumours

The main rumour reported to result from IRS is infertility, especially for younger men. There were beliefs that the spray “reduces male sexual capacity”, and one respondent even went so far as to say that the government is trying to limit the fertility of Tanzanians. Fertility and large families are very important in much of Tanzanian culture, so this issue is of major concern for some community members:

"*There [are] rumours that these chemicals are bad and cause male sexual capacity to reduce. (IDI/Male/Acceptor/Magu)*"

"*For example my wife is pregnant and they are coming to spray. If she will stop bearing children how will it be? And Tanzania is for bearing children so the nation can grow. (IDI/Male/To be sprayed/Magu)*"

Others thought the spray might be Caucasians’ way of reducing the Tanzanian population:

"*I don’t know now, but don’t they say that they are fumigating in order to reduce us? (IDI/Female/to be sprayed/Magu)*"

While many people were aware of these rumours and misconceptions, not all chose to believe them:

"*That was what hindered [the] insecticide spraying process, they have said that Tanzanians are so many in number they want to destroy us so that few people will remain. That’s not true. I think what caused the people [to refuse] was lack of enough education. (FGD/Women/Rural Musoma)*"

It became clear with more interviews that rumours are a function of time; as the frequency of IRS increases, rumours and misconceptions tend to decline. Interviews with respondents in Bukoba Rural and Urban District, Zanzibar, where IRS has been operational for several rounds, showed fewer reported rumours compared to other sites. The more experience a community has with the exercise, the more those who doubt the process see the benefits with few side effects.

"*Because sometimes during the first days of the round many people refused. But after knowing its importance, everyone, if [they] hear that the sprayers are coming, keeps his properties in order and far away [from the house being sprayed]. They know its importance because not only the mosquito run away but also many other small insects are killed. (IDI/Female/Acceptor/Urban West, Zanzibar)*"

"*Respondent (R): There is a difference because to us who our households were sprayed [in the previous rounds], we see the difference in the sense that the things which we were told they would stop [mosquitoes] have stopped to a greater extent. This is quite different to those people whose households were not sprayed.*"

"*Interviewer (I): Do they [people] say this process brings problems to them?*"

"*R: I do not know. It is only because of their stupidity and lack of education. In the house of the district officer they have done the spraying, what about you? You claim it brings problems. That is being stupid. It has no effects since my child has not died, it only kills chiggers, lice…..what problem do I have then? (IDI/Male/Refuser/Urban West, Zanzibar)*"

#### Quality and quantity of possessions

While not a major concern cited by respondents, quality and quantity of possessions owned may contribute to rejection of IRS. Because the exercise requires moving all of one’s possessions outside of the home, one reason for rejecting IRS was that it would allow neighbours to see all of one’s possessions (and thus level of wealth). There was a fear of being ridiculed by neighbours because of poor quality possessions, which is contradictory to previous research, which showed that community members were fearful of people being envious of their possessions and perhaps stealing from them [10].

"*If they [the sprayers] go in their houses and see the way they live…perhaps is it shameful. Someone doesn’t have a good place to sleep. He/she feels it is not good for one to come into their lives so much. (IDI/male/acceptor/Bukoba Rural)*"

There was also a belief by a few participants that sprayers are “coming to spy on peoples’ lives.”

#### Political propaganda

Since spraying and the start of data collection for this study was being conducted shortly before the national election in October 2010, some respondents thought the spraying and the study was politically motivated in that national political parties and candidates were sponsoring the exercise in order to gain votes. However, this varied between Tanzania Mainland and Zanzibar:

"*For example at [one] ward they were saying that the CCM government [ruling party] people should not accept this, it might have the effects. (IDI/male/refuser/Magu)*"

"*Maybe political difference [is why people refuse]. Yes, maybe it’s because of that but because now we have a government involving all political parties, maybe things will change. (IDI/male/refuser/Zanzibar)*"

#### Logistical difficulties

Not spraying a house may not necessarily mean refusal, but rather that the household was not prepared for the spray, and therefore the exercise was not completed. Or, the suppliers were not prepared with enough chemical or could not complete spraying of all houses in a village:

"*The division leader came. Thus we asked the leader, our things had been out of the house for a while now and no one has come to do the spraying. By the moment we were talking to our leader it was around 4 pm. What he told us was that the ICON used for spraying had finished [sprayers ran out of chemical]. From that moment we have not heard from them again. (IDI/Female/refuser/Bukoba Rural)*"

"*On the fumigation day there was rain, the fumigators ran due to the rain and many houses were not fumigated. The second time at a certain place they said they had run out of insecticide. But they had told us to get prepared. (IDI/Female/refuser/Bukoba Rural)*"

While IRS is supposed to be completely voluntary, it should be noted that perceived fear of government follow up made some refusers pretend to praise the exercise despite logistical concerns so as to escape rebuke from government leaders. Some refusers even pretended that their homes had been sprayed when approached by the research team so as not to look like the minority that had not receiving the spray.

### Reasons for accepting IRS

Acceptance of IRS was logically due to the reduction in insects, reduction in frequency of malaria incidence, and reduction in the number of times medical treatment is required:

"*My children used to be sick frequently, but now am glad…it is almost a year they are not suffering anymore like they used to. (IDI/male/refuser/Bukoba Rural)*"

Past experience with IRS influences acceptance or rejection of the subsequent rounds. In this study, respondents who had a positive experience during previous rounds of spraying were more likely to accept it in the subsequent rounds. Other respondents whose houses were sprayed in the previous rounds but missed the most recent round expressed their regret:

"*Personally, I was very happy because in my house there were bedbugs. Okay, those bed bugs plus others insects were all destroyed by this spraying exercise. But my plea is they did not spray in my house the second time they came. (IDI/male/refuser/Bukoba Rural)*"

Community members also talked about accepting IRS in order to provide health protection to neighbours as well as short and long-term family visitors. They believe that when one house is not sprayed, the mosquitoes from that house could go to the neighbours’ houses and infect those residents.

Compliance to authority without questioning was another reason for accepting IRS, even though it is meant to be voluntary. Some people seemed to accept IRS simply because it is announced by community leaders and specialists. Others accepted because of fear of perceived (though not endorsed by this project) follow up by the government in the form of fines and/or imprisonment should they refuse:

"*We accept because it’s a government order. At the first time they said they are coming to spray, and it’s an order from the government so as to reduce the children deaths. (IDI/female/acceptor/Musoma Rural)*"

"*Why would I refuse while it’s an announcement from the government? (IDI/female/to be sprayed/Musoma Rural)*"

"*It is because I just cannot break the law. (IDI/female/acceptor/Bukoba Rural)*"

Respondents who refused during the first round of spraying soon learned that many of the fears surrounding IRS, particularly those regarding side effects, were rumours. This led to acceptance in subsequent rounds.

"*The second time happened, people had not any problem, because they got information. I mean the drug when it was sprayed for the first time nobody died, no one got side effects, hence when they came to sprinkle, no one was against it.****(****FGD/female/Maluku)*"

Respondents are also more “relaxed” as a result of the exercise in that they do not have to worry about as many insects or seeking medical attention on a regular basis. This is especially important to those in the rural areas who have the least access to medical treatment, and it is often more difficult to get transportation to the clinic or hospital, especially during the rainy season. This leads to continued acceptance.

### Women’s involvement with and concerns about IRS

We thought women would not have much say in the decision-making process about whether or not IRS was accepted due to the patriarchal culture in Tanzania. Often, heads of households in Tanzania, especially in the rural areas in which this study took place, the man is seen as the head of the household and final decision maker for issues that will impact his famly (in fact, we approached the head of household for permission to conduct the interview first and foremost). But it seems as if the decision whether or not to accept IRS was not made exclusively by men. Many women said they were involved in making the decision along with the head of the household, and these women generally supported the spray process. For those women who reported to not have as much decision-making power, there was a fear that a head of household who refuses IRS is putting pregnant women and children at risk unnecessarily.

"*When neighbours do not accept the spraying, the children and pregnant women are most affected. It causes suffering to the children in the house, pregnant women can die because of his arrogance—he [who] refuses to be sprayed. (FGD with Women, Magu)*"

One unique concern regarding IRS for women was difficulty in preparing for the exercise. If a woman is alone with a child on the spraying day, it is often challenging for her to care of children, cook, and prepare the house for spraying. In addition, removal of properties in the house is often perceived to be the duty of the husband, but it is unclear whether this is because of the physical labour involved or that the man and head of the household must oversee such activities. A few women reported that they cannot remove heavy items from the home themselves and so sometimes forego the spraying because the household is not prepared.

"*I wasn’t around I went to a court, so the wife was there with a young child….so it was difficult to take out the stuffs. (IDI/male/refuser/Magu)*"

Overall, women are pleased with the effects of IRS—children are not getting sick as often, and even the women themselves are not contracting malaria, which allows them to continue their duties as housewives and mothers.

"*Frankly speaking many of my relatives are so thankful about the spraying of the insecticides because since the years this health service started, many people realize that the insecticide is helping us. Our children can’t get malaria fever, which was a big problem to our families due to death of children below one year age up to five years. Therefore, we are very thankful….And we are also saying that if this health will continue, we can see how our children are continuing growing in a good health and proceeding well with their studies, as well us pregnant women at home. (FGD with Women, Rural Msoma)*"

As one woman put it, *“Malaria disturbs the children who are under age of five years, and women are the ones who suffer”***(***FGD with Women, Magu).* The spraying also keeps the household free of various insects, which makes cleaning an easier task for women.

### Concerns of fishermen and farmers

Contrary to expectations, there were very few concerns about IRS with regards to the fishing profession. The main concern of this group was that if left over chemical was not disposed of properly, it could be poured into bodies of water and hurt aquatic life. However, like other groups in the communities, the fishermen were most concerned with the spray process, the quality of sprayers, mobilization strategies, and appropriate use of IEC materials. There were no specific concerns held by farmers that the spray may interfere with agriculture. While the farmers we interviewed had heard rumours that the spray may be harmful to crops, they had not seen any evidence of this and did not appear to be concerned.

### Response to IRS from community leaders

Almost all leaders were reported to encourage community members to have their homes sprayed. However, while some participants felt that leaders are encouraging IRS to help the people protect their own health, others felt pressure from leaders to accept. Some leaders were even reported to have threatened people with consequences such as fines if they did not accept the spraying:

"*He [the community leader] comes with the textbook [and says] you are supposed to write in it that there is spraying exercise, and failure to do that will lead to taking strong measures against you. (IDI/male/refuser/Musoma Rural)*"

Views on the role of community leaders in Mainland Tanzania were mixed. Some community members think leaders are not doing much to sensitize the community to accept IRS, while others believe community members should be making decisions that are independent of their leaders’ opinions.

In Zanzibar, community leaders were perceived to be highly concerned with all parts of the IRS exercise, ranging from mobilization of community members to supervision of the actual spraying.

"*[The community leaders] are very strict on this exercise, because if it is your turn, your house to be sprayed, they will come and inspect that the drug has been sprayed. And there is a special form you are required to fill in and given to you. If they will prove that the house is already sprayed they leave. (IDI/Female/refuser/Zanzibar)*"

"*In our society the top officials are the Sheha, and the Shehas have embraced this whole idea whole-heartedly. And since the Shehas are residing in this neighbourhood, what they say have an authority to the people. That is why all do [what] we are told to have a go-ahead from the Shehas. (IDI/NGO Leader/Zanzibar)*"

Several participants in Zanzibar reported that those who refused IRS risk being refused other social services provided in the Sheha’s office, such as getting introduction letters when required, receiving letters that may be sent by friends or relatives *via* the Sheha’s office, and receiving free bed nets, which are distributed in collaboration with the Sheha. Unlike Tanzania Mainland, Zanzibar’s community mobilization for IRS and other issues is influenced by political affiliations:

"*Respondent 1: Others does not agree with orders from Sheha [of the opposition party]…because of their political ideological difference.*"

"*Respondent 2: They [pretend they] don’t know him; they don’t recognize anything and orders from Sheha. So if they see him around they know there is something and they refuse. So they don’t recognize Sheha, so anything from Sheha because he has been given order by Government they don’t recognize it. (FGD/women/Urban West District, Zanzibar)*"

"*There are some people…you know here in Zanzibar is quite different from what happens in Tanzanian Mainland…Many of the people here are modernized. But to the opposition parties they are not in favour of the help from the government, and that are a problem. The Sheikhs and people concerned with the spraying process do struggle with them. You might find that when they go to a house of an opposition party and they end up finding the house is closed, and this is the big challenge that they face. This is common in Zanzibar especially to those people of the other political party. I do not know what will happen now, after having a government of national unity, what is in store as far as this whole spraying process is concerned. (IDI/Male/refuser/Zanzibar)*"

### Thoughts on spraying process

The spraying process itself was viewed very favourably for the most part. There were few complaints about the way the IRS process is handled, other than information ahead of time, as described above. For instance, in Magu, Mwanza, and Musoma Rural, some community members claimed to have not heard anything about the IRS exercise until they saw sprayers in their communities. However, this was not reported consistently because the use of community events to mobilize members described by other participant were thought to be successful.

Specific concerns that imply lack of proper mobilization strategies include lack of information sharing between community leaders and members; use of threats instead of mobilization, such as when leadership would sometimes threaten households with jail or lawsuit if they did not comply; ineffective distribution of flyers (*e.g.*, thrown onto the road); and uncertainties about sprayers’ qualifications because they were not sufficiently knowledgeable about IRS and could not respond to questions. One fisherman said he thought those hired to do the spraying were friends of government officials and, therefore, may not be as qualified:

"*Our government’s problem is that it cares less about people’s well being. If the government was really concerned with this exercise, they would select people who are skilled and capable of executing the spraying tasks. Thus, that act of taking someone who is not qualified simply because you happen to know each other is a sign of lack of concern by the government. (FGD with Fishermen, Musoma Rural)*"

Unlike in previous studies where demographic characteristics of a sprayer influenced the extent of IRS acceptance [10], in this study there was no reported preference in terms of gender of the sprayers. However, community members wanted the recruitment of sprayers to take into account professional qualifications, as well recruitment of sprayers from their own communities.

Some community members complained that the stickers placed on houses to signal whether or not a home had been sprayed were not used consistently to mean the same thing, and the meaning was unclear to households. For instance, red stickers placed on doors were thought by some to mean those whose houses were not sprayed will be arrested. Also, if no one is home when sprayers come, they often do not return, even if the occupant would like the spraying. Thus, the sticker indicates refusal when other circumstances may have prevented the sprayers to reach that particular household.

There were also inconsistencies and confusion in the long-term plan reported by the sprayers. Several respondents mentioned that the spray’s effectiveness lasted for nine months. A fisherman in Musoma Rural said the sprayers told him he could stop using mosquito nets for nine months. Another respondent reported he was not allowed to make any modifications to his house (painting, flooring or decorating) until expiry of the chemical:

"*One thing that I would like to know is that they tell you that, in that paper [flyer], they say if you would have this chemical sprayed …for instance if you have not cemented the floor do not cement it. If you had not painted your walls wait until six months are past. So, I needed to know why do they do that until it is nine months? (IDI/male/Acceptor/Bukoba Rural)*"

In Zanzibar, community mobilization around IRS is a joint effort involving NGOs, the Sheha (community leaders at ward level), and household members at large. The messages promoted by all stakeholders are the same, which guarantees consistency and eliminates confusion among community members. It is likely that this joint effort in community mobilization has contributed to reduction of IRS refusals on the island.

### Recommendations from communities

Participants had several recommendations on what should be done to ensure more people accept IRS. The overriding recommendation was providing public education through community mobilization on the importance of the spray. This education should not only include the procedures to be followed to prepare for the spraying, but also more details on how the chemical affects insects, animals, and people, and what are the long term outcomes (both negative and positive).

"*If this exercise was to be done in the next six months then I would advise them to provide education to people so that an ordinary citizen will know what is actually done and for what purpose. They SHOULD be transparent in all they do so that people will have faith in them. (FGD/Fishermen/Musoma Rural)*"

Some participants who were frustrated by their neighbours’ refusal to participate believed those who refused should be forced to comply in order to protect others in the community from malaria:

"*We think something has to be done to them [those who refuse] as a punishment. This is [a] government chemical, and the government can’t bring something to kill the citizens. It is brought to reduce diseases. If he [a community member] refused, the leaders [of the village] have to take action. (IDI/male/acceptor/Bukoba Rural)*"

Other suggested recommendations included: 1) ensuring the recruitment of honest and trustworthy sprayers; 2) having effective training of sprayers so that they, themselves, have a high level of knowledge that can be passed on to the community during the spray process; 3) ensure that effectiveness of IRS is not compromised by using too much water to dilute the chemical; 4) allow enough time for community mobilization and actively involve community leaders in the mobilization process; 5) monitor sprayers for strict adherence to the prescribed procedures; 6) conduct general community meetings in addition to distribution of IEC materials, which may not be read, especially by those with low literacy levels.

## Discussion

Results of this study show that refusers of IRS in Tanzania tend to be more knowledgeable people such as teachers, drivers, extension workers, and other civil servants who do not simply follow the orders of the local government or the sprayers, but are skeptical about the process until they see true results. It seems as if community members have a basic understanding of IRS, however, it will take more in-depth education of the more skeptical members to decrease the refusal rate. Refusal took three forms: 1) refusing partially until thorough explanation is provided; 2) accepting spray to be done in a few rooms only; and 3) refusing outright. There were very few cases of total refusal in which people vehemently admitted refusing the spray. In most of the refusal interviews, participants justified why their houses were not sprayed, often without admitting that they had refused. Reasons for refusal included initial ignorance about the reasons for IRS, uncertainty about its effectiveness, increased prevalence of other insects, potential physical side effects, odour, rumours about the chemical affecting fertility, embarrassment about moving poor quality possessions out of the house to be seen by neighbours, logistical difficulties, and belief that the spray was politically motivated.

The results of the current research, while specific to communities in Tanzania, produced some themes that are highly consistent with previous findings in other countries.

### Logisitics of IRS implementation

Logistically, local recruitment of sprayers [11] given proper training and education, financial incentives for sprayers in terms of salary or honorariums, good working partnerships with local health officials, and community education and mobilization all appear to contribute to acceptability of IRS [12-14]. One of the factors leading to successful eradication of malaria in Taiwan in the 1960s is said to be the carefully delivered health messages and information that were disseminated before spraying was undertaken [15]. Community members were given incentives for reporting malaria cases and their efforts, and photographs were published in local newsletters [15]. Reports of IRS interventions by the MENTOR Initiative in crisis-affected areas in Kenya, Chad, and Central African Republic demonstrate how sprayers were locally recruited and trained in spraying and disseminating health messages. Further, the community received an explanation of the benefits of spraying before it began [12-14]. This likely played an important role in achieving very high rates of spraying coverage that often exceeded the set targets. Many of these same facilitators and barriers to successful implementation also appeared in the current study. Taken in conjunction with findings from other countries, perhaps a stronger focus on logistics and uniform training and education could further increase acceptance rates in Tanzania.

Lack of information on why spraying is done has been reported both as a potential problem [16] and a barrier to IRS acceptance [17,18], and this study is consistent with this repeated finding. Sprayers often arrive without forewarning and have little ability to give information on why spraying is being done and the benefits of spraying [19-21]. In a study by Montgomery *et al.*[16], people did not understand why IRS was conducted but accepted it. Other studies have found that many respondents object to spraying since they do not understand how it works [17,18]. Many households believe that spraying is not effective in preventing malaria [18]. Some believe that while prior rounds were useful, subsequent rounds of spraying were futile [22]. Others believe that the insecticide solution is too diluted to be effective [22]. These are all consistent with findings in the current research.

This study showed that a minority of participants did not appreciate the work of the sprayers themselves, sometimes believing they had diluted the chemical, had gotten the job without being well qualified, or were not knowledgeable enough to be implementing such an important activity. Other studies have also shown the conduct of sprayers to be questionable. This was observed in a study in Thailand, which detailed how sprayers stole household possessions; spray team leaders tried to sell anti-malarial medicines while on spraying rounds; did not object to incorrect dilution of DDT by sprayers; and behaved rudely towards villagers [20]. A previous study in Dar es Salaam and Tanga Tanzania found that some respondents complained sprayers avoided spraying in houses that were not located on main roads [22]. While respondents in a study in one province of South Africa were largely satisfied with the conduct of sprayers, some complained that sprayers left a mess and damaged household items [17]. In other studies, it has been reported that there was also a fear that sprayers may make known publicly the value of one’s household goods [10], a fear that was expressed in this study. While dissatisfaction with sprayers in the current study was rare, it usually revolved around suspicion of over diluting the chemical or the sprayers not being sufficiently knowledgeable about IRS.

The issue for women regarding IRS in the current study involved logistics of moving possessions out of the home (which could be heavy) while at the same time caring for children. This is slightly different from gender-related challenges in other studies. For instance, in a review of the impact of vector control on women, it was found that the entry of other males into domestic space during times when men of the household are away may be objectionable to some [10]. In that study, when young boys were sent as spray personnel, they were allowed into homes. However, when an adult male sprayer was sent, he was denied entry. The participants in the current study expressed no preference for gender of the sprayers, only the logistics of preparing for the spray was of concern for women.

### Insect prevalence

There was some inconsistency in the belief that IRS actually kills insects in the current study, as some participants reported seeing fewer after a round of spraying, while others saw an increase in certain critters. Other research has shown related inconsistencies. A study in Chiapas, Mexico found that 83.7% of respondents perceived that spraying was beneficial in reducing mosquito bites and cockroaches [19]. However, community resistance on account of a perceived increase in bedbug and/or cockroach population or the activity of such insects has been repeatedly reported [18,20,22]. In Surinam, the increased number of cockroaches started attacking crops and biting children [22]. Rafatjah [23] reported that the chemicals used in IRS may make bedbugs irritable and increase their mobility, giving the false impression that infestation has increased. Further, over time bed bugs may become resistant to the chemicals and reappear. Residents might object to spraying if they think that spraying is a cause of the bed bug problem [23]. A study on the size and life stage structures of heavy infestations of bed bugs in Zulu huts in South Africa also reported that people whitewash walls after DDT spraying since it is perceived to increase biting by bed bugs [24]. A review of malaria control in India has noted that householders are “definitely suffering from high populations of bed bugs resistant to DDT and BHC” [25]. This confusion as to why some bugs appear after spraying found in studies in other countries is consistent with the current research. Perhaps making community members more aware of the types of insects that may become more visible after spraying (and why) would eliminate beliefs that the spray is ineffective at reducing malaria-carrying mosquitoes.

### IRS toxicity

Unlike Tanzania, objection to IRS activities in other parts of the world could be related to the use of DDT, a synthetic pesticide that, while highly effective, is controversial because of its potential negative impacts on agriculture and the environment. The odour of DDT has also been reported to be objectionable [13,19,21]. While there were no claims that ICON used in Tanzania affected agriculture or the environment (participants only heard rumours of this), odour was an issue that arose occasionally in the interviews.

### IRS and politics

Some studies have found that acceptability may not always be related to perceived effectiveness of spraying at all and is instead linked with deference to government authorities or politics. A study in Mozambique found that IRS was generally acceptable and generated a positive response from both householders and health care professionals. The respondents, however, did not perceive IRS to be effective against mosquitoes and believed that the effects were short-lived or non-existent. Rather, acceptance was based on a sense of patriotism, citizenship, a belief in good intentions of local politicians and leaders, and perceived obligation to accept government initiatives that were meant for the good of the people [16]. Some expressed that refusing spraying would amount to ingratitude. Respondents thus opined that they were in favour of more spraying. An observational study in Thailand had similar findings such that the poor generally accepted spraying since they felt obligated to comply with sprayers, who they thought were government officials, while people of higher socio-economic status were more likely to resist spraying [20]. This is highly consistent with the current study, where many participants expressed fear that there would be negative retribution by authorities if they did not comply with the spray process.

Political motivations, or the perception of such, was a recurring theme in the current study. This was also shown to be a concern among Maroons in Surinam, where resistance to sparying was associated with intra-clan rivalry, and distrust was prevalent when sprayers were from a different clan and perceived to be more well-off [18]. In some places, sprayers were recruited exclusively on the basis of clan or religion. Barnes and Jenkins [18] noted that by resisting spraying, householders sought to reduce the salary that would be earned by the sprayers who were not from their clan. Not seeking blessings from local chiefs before initiating spraying was also perceived as a sign of disrespect. The political issues related to spraying in Tanzania seem to be more focused on the belief that political parties are sponsoring the spraying in order to win votes, which could have been exacerbated by the fact that some of the regions in which this research took place were receiving their first round of spraying around the 2010 national election.

It seems as though rejection of IRS, while rare, could be addressed by focusing more on strategic community mobilization and education. With more awareness as to the barriers that exist to acceptance of IRS as outlined in this study, those obstacles could be easily overcome with targeted informational and educational communication.

### Recommendations for moving forward with IRS

The primary recommendation for improving IRS uptake is more comprehensive education of the communities. RTI International subcontracted NGOs and community based organizations (CBOs) to implement IEC activities in the districts. The government played a major role in the selection of NGOs/CBOs in collaboration with RTI and also provided supportive supervision and guidance to them. The NGOs were given funds by RTI to conduct all IEC trainings and advocacy meetings from the district down to the hamlet level. Community leaders (local government) worked hand in hand with NGOs/CBOs to make sure they reached community members with correct information about IRS and malaria in general. But with all of these players involved, it is possible that a more streamlined education process to the communities would be more effective. Perhaps general information meetings could be held focused entriely on IRS, and IEC materials could be distributed, but then NGOs could host follow up meetings to answer additional questions from those who are still skeptical or want more information. The data from this study show that refusal of IRS is usually not just a matter of community members being disagreeable, but rather a misunderstanding or lack of understanding of the process and long-term effects of the exercise. It seems as if a follow-up to initial educational sessions or materials as needed would ensure greater acceptance.

There should also be a focus on encouraging community leaders to educate their constituents rather than threaten them with punishments for noncompliance. Improper involvement of local governmental leaders in community mobilization in IRS appears to have led many to accept spraying not voluntarily, but as part of their civic duty, which mirror findings from studies in Mozambique and Thailand [16,20]. As a result, people’s compliance with IRS should not be equated with people’s acceptance of IRS. Threats by community leaders may have contributed greatly to the reported IRS acceptance. The implications of this passive as opposed to voluntary compliance may in the future encourage people to circumvent the spray process so as to satisfy the community leaders’ demands, either by lying about whether their homes have been sprayed or purposely fleeing the area during spraying days so as to make their homes unavailable. The data from the current study already show these tactics are taking place.

Also important to note is that NGOs utilize different strategies to mobilize communities on IRS acceptance. If these strategies were standardized to some extent (*e.g.*, NGOs had to hold a certain number of community meetings and cover a prescribed agenda) and engaged more closely and appropriately with local authorities, refusal rates may decrease.

Finally, given the changing malaria situation in the country due to IRS, continued efforts are needed to emphasize the benefits of maintaining concurrent use of multiple methods of preventing malaria through community health promotion. A separate study to investigate how nets are being used in homes, whether they are used consistently in sprayed homes, who uses which nets (old or new), and how old nets are disposed of, as all of these factors relate to the introduction of IRS, would contribute to improving coordination of malaria prevention programmes in Tanzania.

### Limitations

Being of qualitative design, this study did not seek to generalize findings to the districts/regions or social/cultural groups that participated in the study. Instead, it aimed to provide highlights on the contextual issues that may have a bearing on the acceptance or rejection of IRS in Tanzania.

Another limitation in this study was the definition of concepts: acceptor and refuser. There was no clear dividing line between the acceptor and refuser, and “refusal” quickly became a gray area. Very few individuals declared themselves “refusers”. A majority of people who refused IRS (as indicated by the red sticker on their doorsteps and confirmed by community leaders) did not classify themselves in that way, and this affected the mode of asking questions. As a solution to this, the questions to a refuser in an interview were often posed by referring to the third person (*i.e.*, seeking views or opinions of the interviewee about refusers in his/her community instead of asking for one’s personal opinion). For instance, of all the refusers interviewed, none answered a direct yes to the question, “Did you refuse to have your house sprayed?” Refusers tended to be highly suspicious of the research team, sometimes equating them with a government mission to follow up on IRS refusers. This mistrust between the study team and the community leaders on the one hand and refusers on the other hand indicates a lack of intensive community mobilization.

Interpretation of findings on IRS in Zanzibar deserves special attention. With its low malaria incidence and shift to targeted spraying, Zanzibar experiences specific needs and strategies so as to maintain acceptance of IRS. Findings indicate that formerly, IRS acceptance and rejection were divided politically in Zanzibar. Now that the country underwent referendum in 2010, political differences were not among the reasons for refusal of IRS. In fact, views from community and NGO leaders interviewed in the current study indicate that IRS refusals in Zanzibar are fewer than ever before, despite the shift from blanket spraying to targeted spraying. Important also to note is how Zanzibar has shifted away from treating malaria interventions (*e.g.*, case management, malaria in pregnancy, vector control and community mobilization) as disconnected efforts. In the Zanzibar data from this study, it was evident that community members stress the importance of these interventions simultaneously and sometimes concurrently. It can therefore be tentatively concluded that as the level of malaria transmission decreases, emphasis on implementing several interventions concurrently becomes imperative.

Timing of data collection may also have influenced results. A majority of the data was collected at the beginning of the rainy season before prevalence of mosquitoes visibly increased. As the rainy season progressed, it is possible that benefits of IRS were seen more clearly by communities. Data collection also began around the 2010 National Election. The research team was sometimes unknowingly associated with political parties rather than independent evaluators of IRS, which may have influenced participants’ willingness to be honest about their experiences with the spraying process.

## Conclusions

Overall, IRS has been widely accepted by Tanzanian communities and is being well received as the benefits to communities are becoming more apparent, however, there are still community members who are opposed to IRS or have decided for the time being that they will not accept the treatment. Those who are refusing (mostly in a passive manner), are more educated members of society who are skeptical of IRS’s effectiveness or are suspicious of the motivations behind the spray. With more emphasis during mobilization actitivies on what households can expect during the spray process and for the months following it, who the players are involved in IRS implementation and why, and follow-up with community members that require further information, the minority who refuse IRS may begin to feel more comfortable with the exervise. Using the findings from this research in community mobilization and continued sensitization throughout the IRS process is important to maintain community support for the exercise. It is only through further tightening of mobilization processes and implementation coordination of IRS roll-out that Tanzania will continue to see large scale acceptance of this prevention strategy and a future free of malaria.

## Competing interests

The authors declare that they have no competing interests.

## Authors' contributions

MK designed the study and interview guides, analyzed the data, and drafted the manuscript. DR contributed to the interview guides, supervised data collection, analyzed the data, and helped to draft the manuscript. HK provided feedback on the interview guides and contributed to parts of the manuscript. JM oversaw all data collection and contributed to parts of the manuscript. All authors read and approved the final manuscript.
